# Equine Parvovirus-Hepatitis in China: Characterization of Its Genetic Diversity and Evidence for Natural Recombination Events Between the Chinese and American Strains

**DOI:** 10.3389/fvets.2020.00121

**Published:** 2020-03-10

**Authors:** Gang Lu, Liyan Wu, Jiajun Ou, Shoujun Li

**Affiliations:** ^1^College of Veterinary Medicine, South China Agricultural University, Guangzhou, China; ^2^Guangdong Provincial Key Laboratory of Prevention and Control for Severe Clinical Animal Diseases, Guangzhou, China; ^3^Guangdong Technological Engineering Research Center for Pet, Guangzhou, China; ^4^Guangdong Laboratory for Lingnan Modern Agriculture, Guangzhou, China

**Keywords:** equine parvovirus-hepatitis, horses, China, natural recombination, genetic diversity

## Abstract

Equine parvovirus-hepatitis (EqPV-H) was first reported in a horse that died of equine serum hepatitis in the USA in 2018, and was determined having a strong association with equine serum hepatitis in the following studies. As a newly discovered virus, the genomic sequences of only seven EqPV-H strains have been reported. Considering this, an epidemiological study was performed to investigate the prevalence of EqPV-H in equines in Guangdong Province in China, and obtain genomic sequences of the field prevalent EqPV-H strains. The detection rate of EqPV-H was finally determined to be 8.33% (95% CI: 2.8–18.4%), and EqPV-H's coinfection with equine hepacivirus and equine pegivirus was also determined. Then, the genomes of the Chinese field EqPV-H strains were obtained by PCR, sequencing, and assembly. Through bootscanning analysis, Simplot analysis, and phylogenetic analysis, strong evidence for natural recombination events were found in two Chinese field EqPV-H strains. The natural recombination events occurred between the Chinese and American strains, and were determined within VP protein. Finally, the genetic distance of EqPV-H strains was investigated. Nucleotide identities of 97.1–99.9% and 95.2–100% were found for NS and VP between EqPV-H strains, respectively. Together with other molecular evidence obtained in the present study, the genetic diversity of EqPV-H was determined. Taken together, the results of the present study expand our knowledge on the epidemiological characteristics, genetic variability, and evolution of EqPV-H.

## Introduction

Equine serum hepatitis (also known as Theiler's disease, acute hepatic failure, acute serum hepatitis, idiopathic acute hepatic disease, or postvaccinal hepatitis) is considered to be one of the most common causes of acute hepatitis and liver failure in horses. This disease was first described in 1919 by Arnold Theiler and has often been associated with administration of equine blood products (1). After its first report, the possible causative agent of equine serum hepatitis remained unknown for nearly a century until two novel viruses [Theiler's disease-associated virus (TDAV) and equine parvovirus-hepatitis (EqPV-H)] were recently identified using a high-throughput sequencing method (2, 3).

TDAV (classified in *Pegivirus D*) was first determined during an outbreak of equine serum hepatitis in the USA in 2013 (2). TDAV RNA was detected in affected horses and in administered antitoxin samples; therefore, TDAV was considered as the possible agent of equine serum hepatitis. However, this virus has only been observed in Brazilian equines since its first identification (4). EqPV-H was first discovered in a horse that died of equine serum hepatitis in the USA in 2018 (3). Animal experiments have indicated that equines administered tetanus antitoxin contaminated with EqPV-H could develop clinical signs of equine serum hepatitis. TDAV RNA-positive samples from the first report were then reanalyzed, and all of them also contained EqPV-H DNA. Later, in 2018, two prospective studies on equine serum hepatitis were published by the same research group to understand the prevalence of TDAV, EqPV-H and two other emerging equine viruses, equine hepacivirus (EqHV, classified in *Hepacivirus A*) and equine pegivirus (EPgV, classified in *Pegivirus E*), in the confirmed 28 cases of equine serum hepatitis (5, 6). The results demonstrated that EqPV-H DNA was present in 27/28 cases and that EqPV-H was strongly associated with equine serum hepatitis. No TDAV RNA was detected in any of the cases. These preliminary data demonstrated the causative association between EqPV-H, rather than TDAV, and equine serum hepatitis.

Genetic recombination events are frequently reported in viruses. Recombination can combine mutations from different organisms within a single genome and is considered to play an important role in the genetic diversity and evolutionary patterns in many virus families. Parvoviruses are small single-stranded DNA viruses that can infect a wide range of hosts, including humans and domestic and wild animals (7). Parvovirus genomes typically include at least two open reading frames encoding a non-structural protein and a capsid protein. The growing family *Parvoviridae* currently comprises 13 genera, as recommended by the International Committee on Taxonomy of Viruses (ICTV) (8). A number of recombination events have been reported in parvoviruses, such as human parvovirus B19 (9), bocavirus (10), bufavirus (11), porcine parvovirus (12), Muscovy duck parvovirus (13), goose parvovirus (14), and canine parvovirus (15). As EqPV-H is the newest member of the parvovirus family, whether recombination events occur within EqPV-H strains is still unknown.

Guangdong Province, located in southern China, has the largest number of racehorses and equestrian training centers in mainland China. In this study, the prevalence of EqPV-H was investigated in racehorses in equestrian training centers with no history of equine serum product treatment in Guangdong Province with the aims of (i) studying the prevalence of EqPV-H and other emerging equine bloodborne viruses in horses without preceding equine blood products, (ii) investigating the genetic diversity of EqPV-H based on the sequenced genomes of the field-prevalent strains identified in the present study, and (iii) detecting whether recombination events occur within EqPV-H strains.

## Materials and Methods

### Sample Collection

In December 2018, a total of 60 blood samples were collected from racehorses in an equestrian training center located in Guangdong Province, southern China. The horses at this equestrian training center had been routinely vaccinated for equine influenza (Proteqflu Te, France) and Japanese encephalitis (Nisseiken, Japan) but had not been treated with any equine blood products. After collection, the blood samples were immediately transported to our laboratory in an icebox. Then, the serum was separated by low-speed centrifugation at 3,000 × g for 10 min at 4°C. Two hundred microliters of serum was analyzed for liver enzyme activity and total bilirubin (TBIL) levels, and the remainder of each sample was stored at −70°C for further use.

### Liver Profile

Liver injury in 45 racehorses was evaluated by measuring the enzymatic activities of gamma-glutamyl transferase (γ-GT), alkaline phosphatase (ALP), alanine aminotransferase (ALT), and TBIL levels using an Animal Liver Panel (FUJI DRI-CHEM slide; Fujifilm Co., Tokyo, Japan) with a clinical chemistry analyzer (FUJI DRI-CHEM 700i for veterinary use; Fujifilm Co., Tokyo, Japan). A detected value above the upper limit of the reference range was considered an elevated value.

### Virus Detection

Viral RNA/DNA contained in 50 μl of each of 60 serum samples was processed for nucleotide extraction using a MiniBEST Viral RNA/DNA Extraction Kit (Takara, Japan) following the manufacturer's instructions and then diluted in 30 μl of RNA/DNA nuclease-free water. Six microliters of the extracted RNA was then subjected to cDNA synthesis using a HiScript II 1st Strand cDNA Synthesis Kit (Vazyme, Nanjing, China) with random primers as the reverse transcription primers. As performed in our previous studies, nested PCR (targeting the EqPV-H VP gene/EqHV NS3 gene) and two rounds of RNA PCR (targeting the EPgV NS3 gene/TDAV NS3 gene) were performed using GenStar Taq Polymerase Premix (Kangrun Chengye, China) with the corresponding PCR procedures and primers.

After PCR, the products were detected by electrophoresis on 1.5% agarose gels. Products with the expected PCR band were considered positive samples. The nucleic acids contained in the agarose gel were then purified using a universal DNA purification kit (Tiangen, China). The purified nucleic acids were sent for direct Sanger sequencing from both ends using the PCR primers for detecting the corresponding viruses (BGI, China). After obtaining the raw sequencing data, the presence of the detected equine viruses (EqPV-H, EqHV, EPgV, and TDAV) was assessed with the online BLAST analysis tool (https://blast.ncbi.nlm.nih.gov/Blast.cgi).

### Viral Genomic Sequencing and Analysis

To obtain the genome sequences of the EqPV-H strains in this study, three independent PCRs were performed using the overlapping primer pairs designed in our previous report (1104F, R; 1609F, R; and 2508F, R), which match well with all known EqPV-H strains worldwide. The viral genomic fragments were PCR-amplified using Phanta Max Super-Fidelity DNA Polymerase (Vazyme, China). The amplified products were then purified, and the DNA concentration was determined with a NanoDrop (Thermo, USA). The DNA fragments were ligated into pCloneEZ vectors (Clone Smarter, United States), and the recombinant plasmids were transformed into *E. coli* DH5α competent cells (Weidi, China). The positive *E. coli* culture was sent for sequencing (BGI, China). The genome was assembled with SeqMan 7.1.0 based on the raw sequencing data.

The genome sequences of the field EqPV-H strains were then aligned with those of the American EqPV-H strain BCT-01 and other viruses classified in the genus *Copiparvovirus* with the clustalW method using BioEdit 7.0.9.0. The nucleotide and protein identity between strains was determined and displayed using MegAlign 7.1.0.

To understand the evolutionary relationships between the Chinese EqPV-H strains and other parvoviruses, NS protein sequences of parvoviruses were obtained from the online GenBank database (https://www.ncbi.nlm.nih.gov/genbank/). After alignment and estimation with the “Find Best Protein Models” program, a maximum likelihood (ML) phylogenetic tree based on the NS proteins of the parvoviruses was inferred with the General Reverse Transcriptase (rtREV) with Frequency (+F) and Gamma distributed with Invariant sites (G+I) substitution models and 1,000 bootstrap replicates using MEGA 5.05.

### Recombination Event Analysis

The genome sequences of the EqPV-H strains found in China and the USA were aligned with the clustalW program using BioEdit 7.0.9.0. To detect whether recombination event(s) occurred in EqPV-H strains, the alignment was tested for recombination by seven methods (RDP, GENECONV, Chimera, MaxChi, BootScan, SiScan and 3Seq) using the Recombination Detection Program (RDP) version 4.27. The level of significance was set to *p* < 0.01, and a recombination score of >0.6 was considered to indicate a possible recombination event. In addition, the break points of each recombination event were determined. Then, the recombination event was further determined with a standard similarity plot using SimPlot 3.5.1., with a window size of 1,000 bp and a step size of 100 bp. Finally, maximum likelihood (ML) phylogenetic trees based on the recombinant and non-recombinant VP genomic regions of the EqPV-H strains were generated using MEGA 5.05 with 1,000 bootstrap replicates.

## Results

### Virus Detection in Horses

Equestrian training center H is located in Guangdong Province, southern China, and owns the largest number of racehorses in this province. To understand the epidemiological status of EqPV-H in racehorses in equestrian training center H, serum samples were collected from 60 animals (H1-H60). The average age of the tested animals was 13.1 years (ranging from 3 to 21 years). The majority of these animals were geldings (29/60) and thoroughbreds (25/60).

After analysis by nested PCR and agarose gel electrophoresis, five serum samples were ultimately determined to yield the expected band size. The following online BLAST analysis demonstrated that the sequencing data for all five serum samples produced hits for EqPV-H. This finding indicated that EqPV-H was circulating in the tested racehorses, with a rate of detection of 5/60 (8.33%; 95% CI: 2.8–18.4%) (Table 1). EqPV-H infection was observed in 1 Arabian horse, an 11-year-old stallion, and 4 thoroughbreds, including a 14-year-old gelding, a 21-year-old gelding, another 21-year old gelding, and a 12-year-old mare; these five field EqPV-H strains were named H18, H29, H31, H40, and H46, respectively. In addition, the serum was tested for three other emerging equine bloodborne viruses (EqHV, EPgV, and TDAV) to investigate possible coinfection of these viruses with EqPV-H. The PCR, sequencing, and BLAST analysis results indicated that the detection rates of EqHV, EPgV, and TDAV were 19/60 (31.7%; 95% CI: 20.3–45.0%), 3/60 (5.0%; 95% CI: 1.0–13.9%), and 0/60 (0.0%; 95% CI: 0.0–6.0%), respectively. Two racehorses that tested positive for EqPV-H were found to be coinfected with EqHV (male, 12 years old, thoroughbred) or EPgV (gelding, 14 years old, thoroughbred) (Table 1).

**Table 1:** Information on the EqPV-H positive-equines, results of viral screening, liver enzyme activity, and total bilirubin levels.

**Equine number**	Sex^*^ M/F/G	**Average age^#^**	**Breed**	**EqPV^§^**	**EqHV^§^**	**EPgV^§^**	**TDAV**	**TBIL^a^ (μM/L)**	**γ-GT^b^ (U/L)**	**ALP^c^ (U/L)**	**ALT^d^ (U/L)**
H18	S	11	Arabian horse	**+**				37	18	140	**16**
H29	G	14	Thoroughbred	**+**	**+**			32	13	131	11
H31	G	21	Thoroughbred	**+**				21	20	169	<10
H40	G	21	Thoroughbred	**+**				38	**32**	143	<10
H46	M	12	Thoroughbred	**+**		**+**		35	14	117	11

* *S/M/G: stallion/mare/gelding*;

# *age in years*;

§ *infection indicated by “+”*.

a *Reference range in horses: 9–51 μM/L*;

b *Reference range in horses: 6–24 U/L*;

c *Reference range in horses: 127–241 U/L*;

d *Reference range in horses: <12 U/L*.

e *Values exceeding the upper limit of the normal range are indicated in bold*.

### Liver Enzyme Activity and Total Bilirubin Level Detection

All of the sampled racehorses in the present study were apparently healthy. To investigate whether EqPV-H infection of racehorses could result in liver injury, AST, GGT, and GLDH activity and TBIL levels in 45 serum samples (21 viral RNA/DNA-positive samples and 24 viral RNA-negative samples) were tested. The results demonstrated that 8 positive (2 EqPV-H-positive, 6 EqHV-positive) and 3 negative animals had elevated enzyme activity and/or TBIL levels. A total of 1, 7, and 4 horses presented elevated TBIL, γ-GT, and ALT values, respectively. Two EqPV-H -positive horses had a 1.3-fold higher ALT and γ-GT activities respectively (Table 1).

### Viral Genomic Sequencing and Analysis

Until now, the genomic sequences of seven EqPV-H strains have been reported, including the six Chinese EqPV-H strains (A3, C11, C14, D4, E35, and E36) described in our previous study and the first American EqPV-H strain (BCT-01) (16). The EqPV-H genome contains two predicted open reading frames coding for NS and VP viral proteins, and their 3′ terminal regions are followed by two intergenic regions (intergenic region 1 and intergenic region 2) (3). In the present study, the genomic sequences of five other field EqPV-H strains were amplified by PCR, sequenced and assembled, including the complete nucleotide sequences of NS (1,779 nucleotides), VP (2,922 nucleotides), intergenic region 1 (21 nucleotides), and partial intergenic region 2 (18 nucleotides). The genomic sequences have been submitted to the GenBank database (accession nos. MN218583-MN218587).

The nucleotide content of each EqPV-H strain was calculated and compared. The G+C contents of the five EqPV-H strains (49.36–49.65%) in the present study were equal to those of other previously reported strains (49.38–49.69%). Compared with previously reported EqPV-H strains in China and the USA, the genome sequences of the strains in the present study exhibited no nucleotide insert/deletions. The nucleotide sequence of intergenic region 1 was considered conserved among EqPV-H strains. However, two unique nucleotide variations (A5C and C21T) were observed in intergenic region 1 of H18 that were not found in other strains (Supplementary Figure 1). This finding indicates a novel genomic characteristic of EqPV-H. In addition, a total of 61 and 107 unique nucleotide substitutions were observed in the NS and VP genes of the five Chinese EqPV-H strains, causing 20 and 38 unique amino acid substitutions, respectively (Supplementary Tables 1, 2). Interestingly, most of the nucleotide/amino acid substitutions were observed in H18 and H29. In contrast, no unique nucleotide/amino acid substitutions were found in the H31 genomic sequence.

Some parvovirus species can encode >1 VP proteins, and their NS proteins are more suitable than the VP proteins for analyzing the relationships between parvoviruses. To determine the relationships between the EqPV-H strains in the present study and other parvoviruses, the NS protein sequences of the viruses were used for phylogenetic analysis (Supplementary Figure 2). As expected, the EqPV-H strains identified in the present study grouped together with previously reported Chinese and American strains in the genus *Copiparvovirus*. Another EqPV (EqPV-CSF) identified in cerebrospinal fluid (CSF) collected from a horse with neurological signs and lymphocytic pleocytosis was also grouped in the genus *Copiparvovirus* but had a distant relationship with EqPV-H.

### Natural Recombination Event Detection

**Figure 1:**
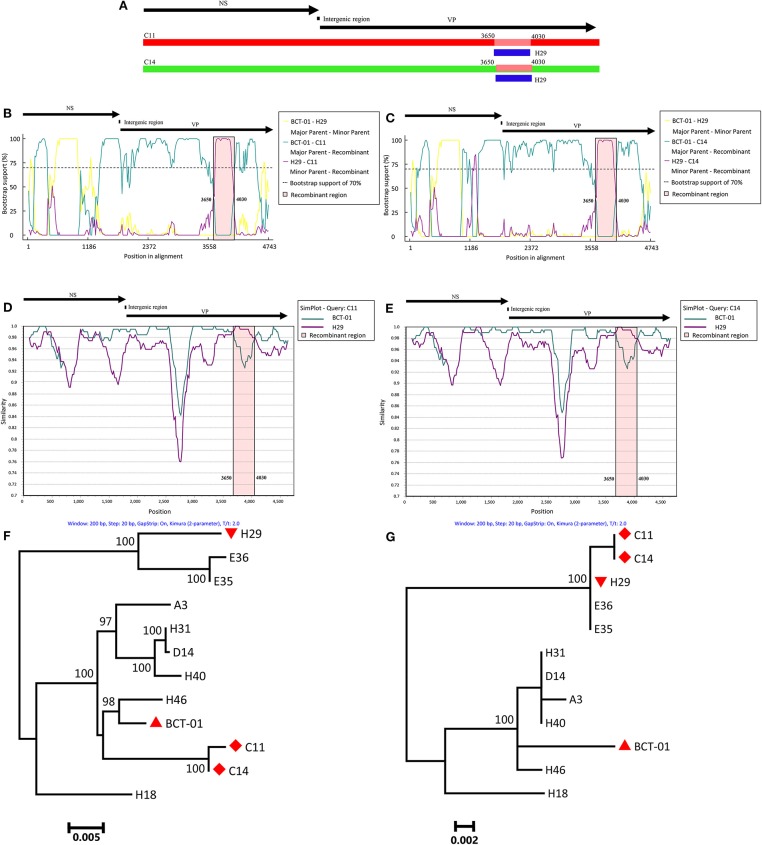
Recombination analysis based on the genomes of C11 and C14. A schematic of the genome organization of EqPV-H is shown above each figure. **(A)** Schematic; the recombinant genomic region of C11 and C14 is indicated in blue. **(B)** Bootscanning analysis of C11. **(C)** Bootscanning analysis of C14. **(D)** Simplot analysis of C11. **(E)** Simplot analysis of C14. **(F)** Phylogenetic analysis of C11 and C14 based on the non-recombinant VP genomic regions. BCT-01, H29, and C11/C14 are indicated by an upright triangle, an upside-down triangle, and a diamond, respectively. **(G)** Phylogenetic analysis of C11 and C14 based on the recombinant VP genomic regions. BCT-01, H29, and C11/C14 are indicated by an upright triangle, an upside-down triangle, and a diamond, respectively.

To investigate whether recombination events occurred between EqPV-H strains, the genomic sequences of all the EqPV-H strains were processed for analysis by seven methods using RDP 4.27. The results demonstrated recombination events within two Chinese EqPV-H strains, C11 and C14, with *p* < 0.01 in all analyses based on the seven methods (Table 2). The major parent-like strain of C11 and C14 was determined to be the American EqPV-H strain BCT-01, and the minor parent-like strain of C11 and C14 was found to be the Chinese EqPV-H strain H29 identified in the present study, with recombination breakpoints mapping to position 3,650 (beginning breakpoint) and 4,030 (ending breakpoint) of the genome, i.e., position 1,850 and 2,230 of the VP gene (Figure 1A).

**Table 2:** The *P*-values for recombination events in the seven detection methods used in this study.

**Method**	***P*****-value**	
	**C11**	**C14**
RDP	2.093 × 10^−5^	2.218 × 10^−4^
GENECONV	7.989 × 10^−6^	7.989 × 10^−6^
BootScan	8.298 × 10^−8^	1.3248 × 10^−5^
MaxChi	1.625 × 10^−3^	1.905 × 10^−3^
Chimera	1.301 × 10^−3^	1.798 × 10^−3^
SiScan	4.760 × 10^−4^	4.760 × 10^−4^
3Seq	3.985 × 10^−4^	3.985 × 10^−4^

Bootscanning analyses also indicated that both C11 and C14 were recombinant strains. When C11 was used as the inquiry strain, the majority of the genome (nucleotides 1–3,649 + 4,031–4,743) of C11 had high bootstrap value support with BCT-01, but nucleotides 3,650–4,030 of the genome had high bootstrap value support with H29 (Figure 1B). A similar phenomenon was also observed when performing bootscanning analyses on C14 (Figure 1C).

Recombination events were further determined by SimPlot analysis. As shown in Figures 1D,E, the entire genomes of C11 and C14 had high genetic similarity with that of BCT-01, except that the nucleotides 3,650–4,030 had high genetic similarity with the sequence of H29.

Two ML phylogenetic trees were eventually constructed to understand the genetic relationships among the recombinant and non-recombinant VP genomic regions of C11, C14 and other EqPV-H strains (Figures 1F,G). In the phylogenetic tree based on non-recombinant genomic regions, C11 and C14 had the closest relationship with BCT-01. However, C11 and C14 had the closest relationship with H29 in the phylogenetic tree based on recombinant genomic regions. In contrast, in both phylogenetic trees, the genetic relationships among most of the other EqPV-H strains were consistent. For example, H29 always clustered together with E35 and E36 in one branch, while BCT-01 had the closest relationship with H46 in the other branch in both phylogenetic trees.

### Nucleotide/Amino Acid Identity Analysis

Because recombination events can interfere with sequence distance analysis, the recombinant strains C11 and C14 were excluded from further nucleotide/amino acid identity analysis.

Compared with each other, the NS coding sequences of the EqPV-H strains had nucleotide/amino acid identities of 97.1–99.9%/96.6–99.8% (Supplementary Table 3), and the VP coding sequences of the EqPV-H strains had nucleotide/amino acid identities of 95.2–100%/95.4–100% (Supplementary Table 4). The lowest nucleotide identities for NS and VP were found between H18 and H29 (97.1%) and between H40 and H29 (95.2%), respectively. In addition, H29 NS and VP had nucleotide identities of 97.1–97.8% and 95.3–96.7% with those of other EqPV-H strains, respectively, indicating that H29 was a genetically divergent strain. It was also found that H31 had the highest nucleotide identity with a previously reported strain, D4, for both the NS (99.9%) and VP (~100%; only 1 nucleotide difference was found) genes. The above identity analysis indicated the genetic diversity of EqPV-H.

## Discussion

Since its first report in 2018 in the USA, EqPV-H infection in horses has been reported in China only by us; we confirmed the presence of EqPV-H in all five tested equine farms in southern China (16). In the present study, EqPV-H DNA was detected in an independent equestrian training center in Guangdong Province, which further revealed the prevalence of EqPV-H in China. Recently, as reported by Meister et al. in 2019, EqPV-H has frequently been detected in commercial equine serum samples, with a detection rate of 11/18 (17). These positive samples have been collected from the USA, Canada, New Zealand, Italy, and Germany, i.e., North America, Europe, and Oceania. Together, the results of the present study support the presence of EqPV-H in Asia and demonstrate that EqPV-H is possibly prevalent in equines worldwide, without apparent geographical restrictions.

EqPV-H is a bloodborne virus that can be transmitted among equines via contaminated equine blood products (3). Therefore, EqPV-H can possibly be transmitted via blood. However, according to the equine owners, the equines in this study were not treated with any equine plasma products. A study on horses in contact with equine serum hepatitis cases and without preceding treatment with equine blood products revealed that EqPV-H infection was strongly associated with acute/subclinical hepatitis in the in-contact horses (6). It is speculated that the transmission route of EqPV-H might include a bloodborne vector and contaminated medical equipment. The detailed transmission mechanism of EqPV-H still needs further investigation.

DNA viruses use cellular polymerase complexes for replication, which replicate DNA genomes in progeny viruses with high fidelity. Compared with RNA viruses (~10^−9^–10^−10^ substitutions/site/year), DNA viruses generally have lower substitution rates (~10^−3^–10^−5^ substitutions/site/year) (18). However, genetic diversity has been characterized in several parvovirus species. Three genotypes of human parvovirus B19 have been found to differ by 10% in the genomic coding region (19). A genetic divergence of 3.3–7.1% was observed between two human bocavirus 2 genotypes (human bocavirus 2A and human bocavirus 2b) (20). In the present study, various molecular data were obtained supporting the genetic diversity of EqPV-H, such as unique nucleotide mutations in intergenic region 1 of H18, a genetically divergent strain (H29), nucleotide identities of 97.1–99.9% in the NS gene and 95.2–100% in the VP gene between EqPV-H strains, and evidence of the occurrence of recombination events in EqPV-H strains.

In the present study, natural recombination events were identified for the first time in EqPV-H strains, and the occurrence of these events was strongly supported by bootscanning analysis, Simplot analysis, and phylogenetic analysis. Coinfection in a cell would provide the opportunity for recombination of different viral genomes, thereby combining them together in a single genome. Viral DNA from >1 parvovirus strain has been detected in an individual animal (21). Interestingly, the recombinant events in EqPV-H occurred between the Chinese and American strains, which were detected in two racehorses on the same farm. One explanation is that frequent international horseraces could create opportunities for close contact and viral transmission between horses from different countries; if an individual racehorse is coinfected with two EqPV-H strains, a natural recombination event might occur. However, it should be noted only one American EqPV-H strain (BCT-01)'s genomic sequence is available, it is still uncertain whether BCT-01 could represent the prevalent EqPV-H strains in the USA. In addition, as epidemiological data on EqPV-H are still very limited in China, whether BCT-01-like EqPV-H strains are circulating in China is unknown. All these questions require more well-designed and large-scaled epidemiological investigations on EqPV-H in equines in China and the USA.

The recombination event in EqPV-H was determined to occur within its VP gene. The VP gene of parvoviruses encodes a capsid protein that is essential for viral absorption, entry into host cells and replication and for production of infectious virus (22). Recombination events within the VP gene have been reported in several parvovirus species, such as between Muscovy duck parvovirus and goose parvovirus (23), between human bocavirus 2 and human bocavirus 4 (24), and within bufaviruses (11). However, the influences of these recombination events on viral pathogenicity have not been investigated. Notably, no liver damage evidence was observed in horses infected with the two recombinant EqPV-H strains and their minor parent-like strain, but their major parent-like strain was detected in a horse that died of equine serum hepatitis. The clinical implications of EqPV-H recombination within the VP gene need to be determined.

In conclusion, EqPV-H was detected in racehorses not previously treated with equine blood products in China. Genomic sequencing and analysis revealed genetic diversity and natural recombination in EqPV-H. The recombination event occurred between the Chinese and American strains. This study will promote understanding of the epidemiological characteristics, clinical implications, and genetic diversity and evolution of EqPV-H.

## Data Availability Statement

The datasets generated for this study can be found in the genomic sequences and have been submitted to the GenBank database (accession nos. MN218583-MN218587): https://www.ncbi.nlm.nih.gov/genbank/.

## Ethics Statement

The animal study was reviewed and approved by South China Agricultural University Experimental Animal Welfare Ethics Committee.

## Author Contributions

SL: conceptualization, writing—review and editing, supervision, and project administration. GL: methodology, software, formal analysis, investigation, resources, writing—original draft preparation, visualization, and funding acquisition. LW and JO: validation.

### Conflict of Interest

The authors declare that the research was conducted in the absence of any commercial or financial relationships that could be construed as a potential conflict of interest.
