# Supplementary material for: Equine Parvovirus-Hepatitis in China: Characterization of Its Genetic Diversity and Evidence for Natural Recombination Events Between the Chinese and American Strains
Source: Front Vet Sci. 2020 Mar 10;7:121. doi: 10.3389/fvets.2020.00121 (PMC7076910; doi:10.3389/fvets.2020.00121)

**Supplementary Table S1 Unique nucleotide/amino acid substitutions in the NS-coding genomic regions of five EqHV strains determined in the present study.**

| **Strain name** | **H18** | **H29** | **H31** | **H40** | **H46** |
| --- | --- | --- | --- | --- | --- |
| **nt/aa variations** |  |  |  | G40A/D14N |  |
|  |  | G48A |  |  |  |
|  |  |  |  |  | G93C |
|  |  |  |  |  | T126C |
|  |  |  |  |  | A162G |
|  |  |  |  |  | C195T |
|  |  |  |  |  | G198C/Q66H |
|  | G203A | G203A |  |  |  |
|  |  |  |  |  | G216A |
|  |  |  |  |  | C240T |
|  | G243A |  |  |  |  |
|  |  | C306T |  |  | C306T |
|  | G333A |  |  |  |  |
|  |  | C334G/L112V |  |  |  |
|  | G357A |  |  |  |  |
|  |  |  |  | C557G/T186S |  |
|  | T745C |  |  |  |  |
|  | A750G |  |  |  |  |
|  | G816A |  |  |  |  |
|  |  |  |  | G828C |  |
|  | A841C |  |  |  |  |
|  | C850G/Q284E |  |  |  |  |
|  |  |  |  |  | C888T |
|  | T954C |  |  |  |  |
|  |  |  |  | T981A |  |
|  | G1047C |  |  |  |  |
|  | G1116A |  |  |  |  |
|  | T1137G |  |  |  |  |
|  | 41170C |  |  |  |  |
|  | C1171T |  |  |  |  |
|  | A1224C |  |  |  |  |
|  | G1288A/V430I |  |  |  |  |
|  | A1419C |  |  |  |  |
|  |  | C1425T |  |  |  |
|  | G1434A |  |  |  |  |
|  |  | T1440C |  |  |  |
|  | C1452T |  |  |  |  |
|  | C1455A |  |  |  |  |
|  |  | C1455G |  |  |  |
|  |  | G1491C |  |  |  |
|  |  | T1512C |  |  |  |
|  | C1529G/P510R |  |  |  |  |
|  |  | C1563T |  |  |  |
|  | T1573G/S522P | T1573G/S522P |  |  |  |
|  | G1602T/S525A | G1602T/S525A |  |  |  |
|  |  | A1615G/T539A |  |  |  |
|  |  | A1628G/N543S |  |  |  |
|  |  | A1630G/T544G |  |  |  |
|  |  | C1631G |  |  |  |
|  |  | G1640C/R547T |  |  |  |
|  | T1651C/S551P | T1651C/S551P |  |  |  |
|  |  | G1653A |  |  |  |
|  |  |  |  | G1667C/G556A |  |
|  | A1687T/T563S | A1687T/T563S |  |  |  |
|  |  | T1690G/S564A |  |  |  |
|  |  | A1696T/I566L |  |  |  |
|  |  | G1718C/S573T |  |  |  |
|  |  | C1722G/D574E |  |  |  |
|  | G1755C |  |  |  |  |
|  |  | G1755A |  |  |  |
|  |  | C1756T |  |  |  |
|  |  | C1764A |  |  |  |
| **Total** | 27/7 | 27/13 | 0/0 | 5/3 | 9/1 |

**Supplementary Table S2 Unique nucleotide/amino acid substitutions in VP coding genomic region of five EqHV strains determined in the present study.**

| **Strain name** | **H18** | **H29** | **H31** | **H40** | **H46** |
| --- | --- | --- | --- | --- | --- |
| **nt/aa variations** |  | C45T |  |  |  |
|  |  | T171C |  |  |  |
|  | T210C |  |  |  |  |
|  | T217C |  |  |  |  |
|  |  | T324C |  |  |  |
|  |  | C405T |  |  |  |
|  |  | G438A |  |  |  |
|  |  |  |  | A473T/K158I |  |
|  | G489A |  |  |  |  |
|  |  | A498C |  |  |  |
|  |  | C516T |  |  |  |
|  | G531A |  |  |  |  |
|  | C622G/L208V |  |  |  |  |
|  |  | C667T |  |  |  |
|  |  | G706A/A236M |  |  |  |
|  |  | C707T |  |  |  |
|  |  | C747T |  |  |  |
|  |  | G758A |  |  |  |
|  |  | C815T/A272V |  |  |  |
|  |  | G820A/G274A |  |  |  |
|  | G821C/G274S |  |  |  |  |
|  | G821C |  |  |  |  |
|  | A825G | A825G |  |  |  |
|  | T833C/V278A |  |  |  |  |
|  | A844G/T282A |  |  |  |  |
|  |  | C845G/T282S |  |  |  |
|  | T855G |  |  |  |  |
|  |  | T864C |  |  |  |
|  | C865G/L289A |  |  |  |  |
|  | T866C |  |  |  |  |
|  | G871A/G291N |  |  |  |  |
|  |  | T873G/ G291E |  |  |  |
|  | C874T | C874T |  |  |  |
|  |  | G882A |  |  |  |
|  |  | A891T/K297N |  |  |  |
|  |  | C892G/P298A |  |  |  |
|  |  | C898T/P300S |  |  |  |
|  |  | G902A/S301N |  |  |  |
|  | T907C/S303R |  |  |  |  |
|  |  | T907G/S303V |  |  |  |
|  | C908G |  |  |  |  |
|  |  | G916C/V306L |  |  |  |
|  | G925A/A309T |  |  |  |  |
|  |  | C932A/A311E |  |  |  |
|  |  | C933G |  |  |  |
|  | A973T/S325C |  |  |  |  |
|  |  | G977C/S326P |  |  |  |
|  |  | A984G |  |  |  |
|  |  | C1001A |  | C1001A |  |
|  |  | C1003G/P335A |  |  |  |
|  |  |  |  | C1010T/S337L |  |
|  |  | A1034G/K345R |  |  |  |
|  |  |  |  | G1040A/R347Q |  |
|  |  | G1046T/R349L |  |  |  |
|  | T1049C/L350P |  |  |  |  |
|  |  | T1049G/L350R |  |  |  |
|  |  | G1061A/S354N |  |  |  |
|  | A1064C/E355A |  |  |  |  |
|  |  | G1110A |  |  |  |
|  |  | A1158G |  |  |  |
|  |  | A1182C |  |  |  |
|  |  | C1194G |  |  |  |
|  | A1207T/T403S |  |  |  |  |
|  |  | C1249T |  |  |  |
|  | C1251A | C1251A |  |  |  |
|  | G1275T | G1275T |  |  |  |
|  | G1281T | G1281T |  |  |  |
|  |  | G1344A |  |  |  |
|  |  | C1383T |  |  |  |
|  | T1419C | T1419C |  |  |  |
|  | C1438A | C1438A |  |  |  |
|  |  | A1440G |  |  |  |
|  |  | T1441A/C481K |  |  |  |
|  |  | G1442A |  |  |  |
|  | C1443T |  |  |  |  |
|  |  | C1443A |  |  |  |
|  | C1444A/L482M | C1444A/L482M |  |  |  |
|  | G1464A |  |  |  |  |
|  |  | C1497T |  |  |  |
|  | G1503T |  |  |  |  |
|  |  | G1518A |  |  |  |
|  | T1533G |  |  |  |  |
|  |  | A1542C |  |  |  |
|  | A1623G | A1623G |  |  |  |
|  |  |  |  | G1725A |  |
|  | A1754G/K585R |  |  |  |  |
|  |  | C1758T |  |  |  |
|  |  |  |  |  | C1787G/T596S |
|  |  | C1797T |  |  |  |
|  |  | G1818A |  |  |  |
|  | T1869A |  |  |  |  |
|  | C1875G |  |  |  |  |
|  | G1893C |  |  |  |  |
|  | A2085G |  |  |  |  |
|  |  |  |  |  | A2133G |
|  |  | A2241G |  |  |  |
|  | A2307G |  |  |  |  |
|  | C2364T |  |  |  |  |
|  |  | C2397T |  |  |  |
|  |  | A2442C/E814D |  |  |  |
|  | G2469C |  |  |  |  |
|  | C2562T |  |  |  |  |
|  |  | A2620C/N874H |  |  |  |
|  | C2461T |  |  |  |  |
|  | C2712T |  |  |  |  |
|  | C2838T |  |  |  |  |
|  | C2844T |  |  |  |  |
| **Total** | 46/14 | 64/22 | 0/0 | 5/3 | 2/1 |

**Supplementary Table S3 Analysis of the nucleotide (upper right)/amino acid (bottom left) identity of the NS-coding genomic region between EqHV strains.**

|  | **BCT-01** | **A3** | **D4** | **E35** | **E36** | **H18** | **H29** | **H31** | **H40** | **H46** |
| --- | --- | --- | --- | --- | --- | --- | --- | --- | --- | --- |
| **BCT-01** |  | 98.4 | 98.9 | 98.8 | 98.7 | 97.8 | 97.5 | 98.9 | 98.7 | 98.8 |
| **A3** | 99.0 |  | 98.7 | 98.9 | 98.8 | 97.4 | 97.4 | 98.7 | 98.0 | 98.5 |
| **D4** | 99.0 | 99.2 |  | 98.9 | 98.8 | 97.8 | 97.5 | 99.9 | 98.4 | 99.6 |
| **E35** | 98.5 | 99.0 | 98.7 |  | 99.7 | 97.8 | 97.6 | 98.9 | 98.3 | 98.8 |
| **E36** | 98.8 | 99.3 | 99.0 | 99.5 |  | 97.7 | 97.6 | 98.9 | 98.3 | 98.7 |
| **H18** | 98.0 | 98.1 | 98.1 | 97.8 | 98.1 |  | 97.1 | 97.8 | 97.4 | 97.7 |
| **H29** | 96.8 | 97.3 | 97.0 | 97.0 | 97.3 | 97.6 |  | 97.5 | 97.3 | 97.4 |
| **H31** | 99.0 | 99.2 | 99.8 | 98.7 | 99.0 | 98.1 | 97.0 |  | 98.5 | 99.6 |
| **H40** | 99.0 | 98.8 | 98.8 | 98.3 | 98.7 | 97.8 | 96.6 | 98.8 |  | 98.3 |
| **H46** | 98.7 | 98.8 | 99.2 | 98.3 | 98.7 | 97.8 | 96.6 | 99.2 | 98.5 |  |

**Supplementary Table S4 Analysis of the nucleotide (upper right)/amino acid (bottom left) identity of the VP-coding genomic region between EqHV strains.**

|  | **BCT-01** | **A3** | **D4** | **E35** | **E36** | **H18** | **H29** | **H31** | **H40** | **H46** |
| --- | --- | --- | --- | --- | --- | --- | --- | --- | --- | --- |
| **BCT-01** |  | 98.2 | 98.0 | 96.8 | 97.0 | 96.8 | 95.4 | 98.0 | 97.9 | 98.3 |
| **A3** | 97.5 |  | 98.6 | 96.7 | 96.9 | 96.8 | 95.6 | 98.7 | 98.5 | 98.0 |
| **D4** | 97.1 | 97.4 |  | 97.0 | 97.1 | 96.6 | 95.3 | 100.0^*^ | 99.4 | 98.0 |
| **E35** | 97.2 | 96.5 | 96.8 |  | 99.3 | 96.4 | 96.7 | 97.0 | 96.8 | 97.0 |
| **E36** | 97.5 | 96.8 | 96.9 | 98.5 |  | 96.6 | 96.6 | 97.1 | 97.0 | 96.9 |
| **H18** | 97.1 | 96.4 | 96.3 | 96.6 | 97.2 |  | 95.7 | 96.7 | 96.6 | 96.6 |
| **H29** | 96.2 | 96.1 | 95.5 | 96.1 | 96.2 | 96.3 |  | 95.3 | 95.2 | 95.4 |
| **H31** | 97.1 | 97.4 | 100.0^*^ | 96.8 | 96.9 | 96.3 | 95.5 |  | 99.5 | 98.0 |
| **H40** | 96.9 | 97.0 | 98.7 | 96.5 | 96.6 | 96.3 | 95.4 | 98.7 |  | 97.9 |
| **H46** | 96.9 | 96.2 | 96.0 | 97.0 | 96.5 | 96.2 | 95.5 | 96.0 | 95.9 |  |

**Supplementary Figure S1 The nucleotide sequence alignment results for intergenic region 1 of EqPV-H.**

The sequences were aligned with the American EqPV-H strain BCT-01, and identical nucleotides at each site are indicated with dots.


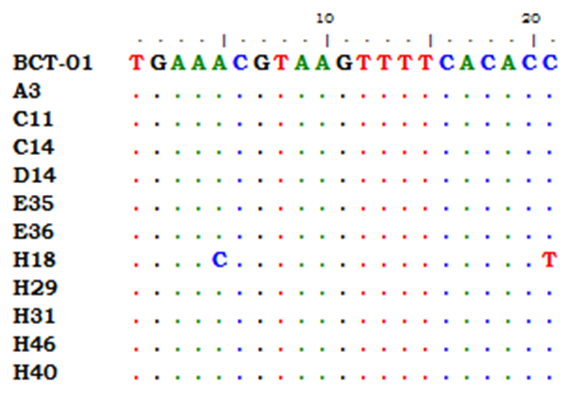


**Supplementary Figure S2 Phylogenetic analysis of the five EqPV-H strains identified in the present study based on the NS protein.**

The EqPV-H strains identified in the present study, the EqPV-H strains identified in our previous study, BCT-01, and EqPV-CSF are indicated with circles, squares, a triangle, and a diamond, respectively.


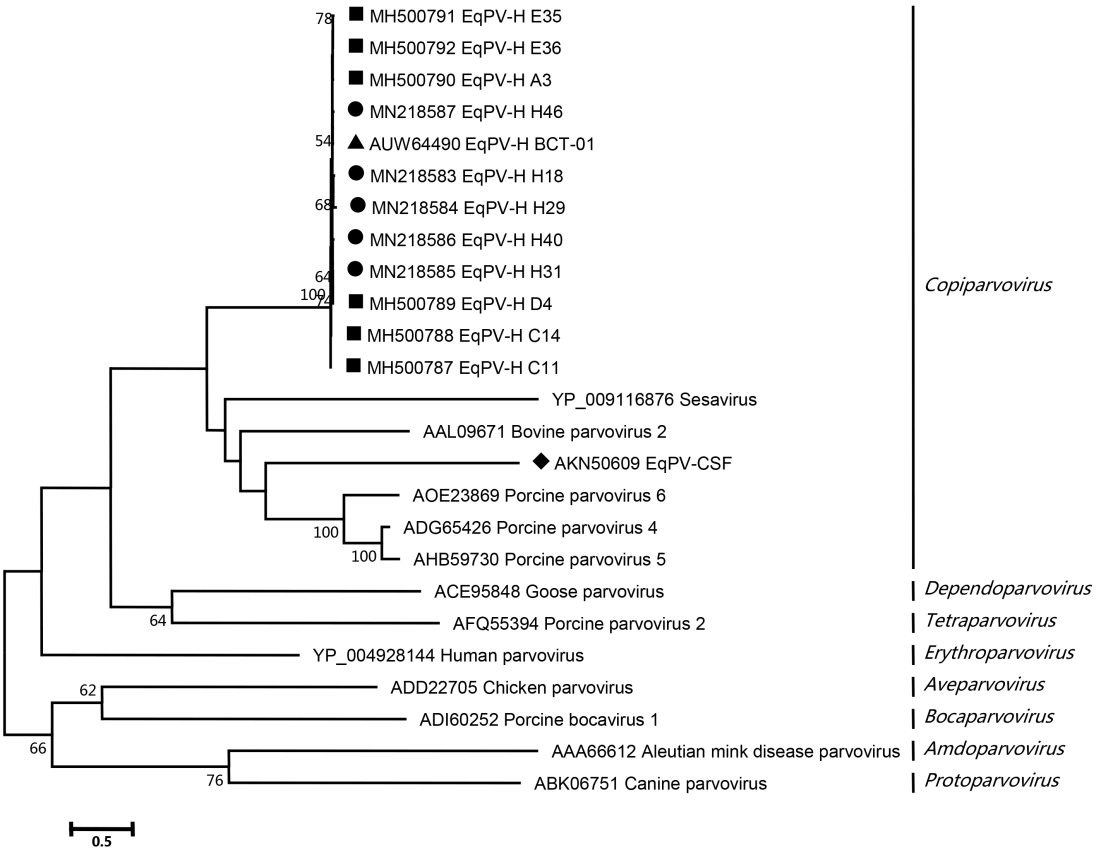

Supplement: Supplementary file 1 [file Data_Sheet_1.docx]
